# Phytochemical Profile and Acute Toxicity in CD-1 Mice of the Hydroethanolic Extract and Butanolic Fraction of *Piper marginatum* Jacq.

**DOI:** 10.3390/jox15050156

**Published:** 2025-09-28

**Authors:** Luis Gonzalo Sequeda-Castañeda, Luis Fernando Ospina-Giraldo, Sandra Janeth Gutiérrez-Prieto, Pilar Ester Luengas-Caicedo

**Affiliations:** 1 Department of Chemistry, Faculty of Sciences, Pontificia Universidad Javeriana, Bogotá 110231, Colombia; 2 Department of Pharmacy, Faculty of Sciences, Universidad Nacional de Colombia, Bogotá 111321, Colombia; 3 Dentistry Research Center, School of Dentistry, Pontificia Universidad Javeriana, Bogotá 110231, Colombia

**Keywords:** *Piper marginatum*, acute toxicity, CD-1 mice, histopathology, metabolomic profile, phytochemical composition

## Abstract

This study evaluated the acute toxicity of the hydroethanolic extract and the butanolic fraction of *Piper marginatum* Jacq., following the OECD Guideline 423. Oral and intraperitoneal exposure of CD-1 mice was used in single-dose and repeated-dose schedules. No mortality or significant behavioral alterations were observed. Body weight remained stable during treatment, and histopathological analysis revealed only mild to moderate alterations, mainly in the liver, kidneys, and lungs. These results indicate the absence of acute systemic toxicity under the conditions evaluated. Preliminary phytochemical analysis and metabolomic profile analysis by LC-QTOF/MS revealed a diverse composition of secondary metabolites, including alkaloids, flavonoids, phenylpropanoids, and sphingolipids. Compounds with known biological activity and some with potential toxicity were identified. The findings support the safe use of *Piper marginatum* extracts in short-term applications and suggest further subchronic toxicity studies and mechanistic evaluation. This research provides fundamental data for preclinical characterization and standardization of extracts of plant origin.

## 1. Introduction

The research on medicinal plants has attracted the attention of the scientific community because of their therapeutic potential. Within this field, the Piper genus stands out for its diversity of secondary metabolites and therapeutic applications. Extracts and fractions of this genus contain flavonoids, chalcones, flavones, phenylpropanoids, lignans, neolignans, terpenes, steroids, piperolides, amides, and alkaloids, which have shown antioxidant, antimicrobial, anti-inflammatory, antitumor, and anxiolytic properties, among others [1,2,3,4,5,6,7,8,9,10,11].

One of these species is *Piper marginatum* Jacq., known as ‘hoja santa’, the soldier’s herb, ‘curadientes’, and ‘cordoncillo’ in Latin America, and is used in traditional medicine to treat inflammation, bacterial infections, gastrointestinal disorders, bleeding, wound healing, and prevent tooth decay [6,9,12,13]. Its extracts, fractions, and essential oils, rich in phenols, flavonoids, phenylpropanoids, amides, and alkaloids, have shown antioxidant, antimicrobial, anti-inflammatory, and remineralization properties in tooth enamel in vitro [6,14,15,16,17,18].

Previous studies carried out by our research groups (Phytochemical Research Javeriana University—GIFUJ, Research in Natural Products Technology—TECPRONA, Dental Research Center—CIO) have shown that extracts and some fractions of *Piper marginatum* have antimicrobial activity against bacteria associated with dental caries (*Streptococcus mutans*, *Streptococcus sobrinus*, *Lactobacillus acidophilus*, and *Lactobacillus casei*) and periodontal diseases (*Porphyromonas gingivalis*, *Prevotella intermedia* and *Fusobacterium nucleatum*) [13,14,15,18]. Furthermore, its ability to promote the remineralization of tooth enamel has been observed in demineralized teeth (induced caries) and in those with imperfect amelogenesis [14,15,18] and with marked activity in the butanolic fraction. Butanolic fractions concentrate specific secondary metabolites, which could increase or decrease their toxicity compared to the total extract [6,7,10,11].

More recently, *Piper marginatum* on mesenchymal stem cells was evaluated, showing a favorable safety profile that supports its pharmacological potential [19]. Taken together, these findings constitute a solid scientific basis that supports the need to move toward preclinical evaluation in standardized animal models and highlights the importance of standardizing extraction procedures to ensure the reproducibility of results [20].

In this context, the use of standardized animal models such as CD-1 mice represents a necessary methodological step within the phytotherapeutic development pathway, as it allows integrating systemic effects, bioavailability, and toxicity in a complex biological environment [21,22]. Other members of the Piper genus have previously been investigated in vivo, showing anti-inflammatory, analgesic, and cytotoxic activities [23,24,25,26,27], confirming the relevance of extending these analyses to *Piper marginatum*. This evidence highlights the importance of bridging pharmacological data in vitro and toxicological validation in vivo.

The proposed experimental design is based on the principle of scientific staggering, advancing from ethnopharmacological observations and tests in vitro to animal models, in accordance with the OECD guidelines and the principle of 3R (replacement, reduction and refinement) [21,28,29]. In this way, the evaluation of *Piper marginatum* extracts and fractions in CD-1 mice addresses not only the validation of a traditional use, but also the construction of a comprehensive scientific framework that combines phytochemistry, experimental pharmacology, and international ethical standards. This transition is essential to establish the preliminary safety and efficacy of the natural resource before proposing subsequent phases of clinical research.

Despite interest in the bioactive properties of *Piper marginatum*, scientific information on its toxicity is limited, which represents an obstacle to its development as a safe therapeutic agent [9,19,30]. Acute toxicity assays, which are essential to determine the mean lethal dose (LD_50_), can identify adverse effects and establish a safety margin for future clinical evaluations [31].

In this context, the purpose of this study was to evaluate the acute toxicity of the total hydroethanolic extract and the butanolic fraction of *Piper marginatum* Jacq. in CD-1 mice, following the OECD guideline 423. This design allows us to integrate previous evidence on its antimicrobial and remineralizing properties, reported by our research group and other laboratories, with toxicological validation in a standardized animal model. In this way, we seek to provide solid data on the preliminary safety of the extracts and correlate them with their phytochemical profile, which is a fundamental step in advancing the pharmacological characterization of the species and the standardization of its extracts for future therapeutic applications.

## 2. Materials and Methods

During the experiments, the ethical, safety and health procedures of the laboratory, the care, and use of laboratory animals were complied with, following the national and institutional standards in force. Additionally, the necessary precautions were taken to minimize the risks associated with the materials and substances used.

### 2.1. Reagents and Equipment

The following Merck Millipore reagents and solvents were used: ethanol (EtOH, 99.5%), hexane (Hex, 98.5%, product number 104374), dichloromethane (CH_2_Cl_2_, 99.8%, product number 106050), butanol (BuOH, 99.7%, product number 101988), deionized water (H_2_O, 100%), glycerin (GL, 99.0%, product number 818709), propylene glycol (PG, 99.0%, product number 803265), paraffin (99.0%, product number 107151), hematoxylin (96.0%, product number 115938) and eosin (95.0%, product number 104134). Equipment such as ultrasound probe (Fischer Ultrasonic, Model 60, Sindelfingen, Germany), rotary evaporator (Büchi, R-100, Flawil, Switzerland), microscope (Zeiss, Primostar 3, Jena, Germany), Shimadzu Prominence liquid chromatograph with UV-Vis detector (Shimadzu Corporation, Kyoto, Japan), Agilent 1200 liquid chromatograph coupled to an Agilent 6520 Q-TOF LC/MS system mass detector (Agilent Technologies Inc., Santa Clara, CA, USA).

### 2.2. Preparation of Plant Material and Extract and Its Fractions

The plant material was donated by Mogambo Sendero Ambiental (Viotá, Cundinamarca, Colombia), without mechanical damage (trauma or defoliation), biological damage (damage caused by herbivores) or microbiological damage (signs or symptoms of phytopathogens), and without soil, insects, or stems. Previously, the plant species *Piper marginatum* Jacq. was taxonomically classified by the Botanist A. Jara of the National Herbarium of Colombia (voucher COL 575454). This research has contracts for Access to Genetic Resources and derived products No. 255 of 2019 (Resolution 0418 of 3 April 2019) and No. 268 of 2019 (Resolution 0841 of 21 June 2019) of the Ministry of Environment and Sustainable Development of the Republic of Colombia (Minambiente).

The dried plant material (leaves, flowers, fruits and young stems) was ground to a particle size between 180 and 355 μm, using a mill with standardized sieves. A total of 50 g of plant material was subjected to ultrasound-assisted extraction (Fischer Ultrasonic, Modelo 60, 50 kHz), using a drug: solvent ratio of 1:30, with an ethanol: water mixture (7:3), for 20 min. This procedure was repeated three times. The three hydroethanolic extracts obtained were combined before concentration. The term total or hydroethanolic extract refers to the crude extract obtained directly from the dry ground plant material of *Piper marginatum*, using an ultrasound-assisted extraction process with a hydroethanolic solution (ethanol/water, 70:30). This extract, which concentrates all the soluble metabolites in this solvent, is used as a starting point for successive partitioning into solvents of different polarity and constitutes the reference matrix for toxicological and phytochemical evaluation. The crude extract obtained was concentrated in a final ratio of 1:1 (mass/volume). Subsequently, a liquid–liquid fractionation was carried out for 24 h, successively using hexane (C_6_H_14_, 100 mL × 3), dichloromethane (CH_2_Cl_2_, 100 mL × 3), butanol (BuOH, 100 mL × 3) and water (H_2_O, 100 mL × 3). The resulting fractions were concentrated by rotational evaporation at 40 °C, lyophilized, and stored at −50 °C in amber bottles [32].

Hydroethanolic extract and the butanolic fraction were used only for toxicity and chemical profiling tests. The butanolic fraction was selected for its enrichment in bioactive metabolites with reported antimicrobial and remineralizing activity and because previous studies in our group showed greater efficacy in in vitro assays.

### 2.3. Experimental Design and Animal Work

#### 2.3.1. Experimental Design

An acute toxicity study was carried out in mice, following the OECD 423 guidelines, in which the morphological and histopathological effects induced by a crude hydroethanolic extract and a butanolic fraction of *Piper marginatum* were evaluated [21]. Substances were administered orally (*po*) and intraperitoneally (*ip*) in single-dose and repeated dose regimens for 14 consecutive days. The design was completely randomized, and treatments were applied under controlled vivarium-based conditions.

#### 2.3.2. Experimental Animals

Swiss albino mice, males and females, with 8.5 ± 0.5 weeks old, nonconsanguineous exogamous group CD-1 (Originating from Charles River Laboratory, Wilmington, MA, USA), with an average weight of 30–34 g (females) and 34–38 g (males), housed in steel boxes with a chip bed, under standardized conditions of temperature (22 ± 2 °C), humidity (55–65%) and light/dark cycle 12:12 h. The animals had access ad libitum to purified water and a standard diet (LabDiet Rodent^®^) [33].

#### 2.3.3. Experimental Groups and Treatments

Mice, both female and male, were randomly divided into treatment groups (*n* = 4) according to the type of substance administered and the route of exposure: control group (vehicle), hydroethanolic extract group (*po*/*ip*) and butanolic fraction group (*po*/*ip*), Table 1. Each treatment was administered at a single dose of 2000 and 1000 mg/kg body weight. (total hydroethanolic extract) and 1000 and 250 mg/kg bw. (butanolic fraction). In the repeated exposure schedule (po for 14 consecutive days), daily doses of 250 mg/kg bw were used for the hydroethanolic extract and 100 mg/kg bw for the butanolic fraction. The solutions were formulated in an aqueous vehicle composed of propylene glycol, glycerine, and water in proportion (80:10:10).

#### 2.3.4. Post-Treatment Clinical and Behavioral Observation

After dosing, mice were observed at minute 0, 5, 15, 30 and 60; then at 2, 4, 6, 12 and 24 h; and then every day for 14 days. Each day the animals were transferred to the observation pen and the Irwin/Hippocratic test adapted to natural products by the Bioterium of Pharmacy of the National University of Colombia was used to record observations in the animals, such as changes in body mass, skin, fur, eyes, mucous membranes, respiratory systems, circulatory, autonomic, central nervous and somatomotor activity and behavior pattern. Similarly, signs such as tremors, convulsions, salivation, diarrhea, lethargy, sleepiness, or other pathophysiological alterations were observed. Animals that showed signs of severe pain or severe distress were sacrificed to avoid suffering [34,35].

#### 2.3.5. Variables Evaluated

The following organs were evaluated: liver, kidney, lung, heart, brain, cerebellum, stomach, small intestine, large intestine, and spleen. The glandular (fundic region) and nonglandular (forestomach) areas of the stomach were evaluated separately. Histopathological variables analyzed included vascular congestion, cell degeneration, inflammatory infiltrates, necrosis, glomerular or alveolar changes, cell death, and other specific structural alterations, depending on the organ [36]. The tissues were fixed in 10% buffered formalin, processed using standard paraffin embedding techniques, and stained with hematoxylin and eosin (H&E). The slides were evaluated by a pathologist, blind to treatment, using a semiquantitative scale for each alteration: 0 = no involvement, 1 = mild, 2 = moderate, 3 = severe or intense [37].

#### 2.3.6. Sampling and Analysis Unit

Each animal was considered an experimental unit. For each organ, at least two representative histological sections were analyzed. Data were organized and consolidated into a matrix that allowed for comparison between organs, treatments, routes, and dose schedules. Descriptive analyses and the calculation of the means by organ and type of exposure were applied [36,37,38].

#### 2.3.7. Ethical Aspects and Method of Slaughter

All procedures were approved by the Institutional Committee for the Care and Use of Laboratory Animals and the Ethics Committee of the National University of Colombia (Acta No. 10 of 23 September 2019). The animals were sacrificed by cervical dislocation, a method approved by the AVMA for rodent euthanasia, carried out exclusively by trained personnel [39]. This technique was selected to avoid the introduction of chemical artifacts that could alter histopathological analyses. However, its possible limitations are recognized in highly vascularized organs, as discussed in the corresponding section [40,41].

### 2.4. Preliminary Phytochemical Analysis

Preliminary phytochemical analysis was carried out following the methodology proposed by Sequeda (2019, 2025) to qualitatively and quantitatively determine secondary metabolites. Tube tests and thin-layer chromatography were used to determine the presence or absence of secondary metabolites such as alkaloids, carbohydrates, coumarins, cardiotonic glycosides, steroids, triterpenes, flavonoids, phenols, naphtho and anthraquinones, saponins, sesquiterpene lactones, tannins, and terpenes. For the quantification of phenols, flavonoids, proanthocyanidins, polyphenols, and tannins, the UV-vis spectrometry technique was used [18,32].

### 2.5. Chromatographic Profiles by HPLC/PDA and LC-QTOF/MS

The HPLC/PDA profile of the hydroethanolic extract was performed using a Shimadzu Prominence liquid chromatograph with the following characteristics: PDA detector (254, 280 and 350 nm). SiliaChrom Pentafluorophenyl Column (4.6 × 250 mm, 5 μm). Mobile phase: solvent A (H_2_O, 0.1% CH_3_COOH), solvent B (CH_3_CN, 0.1% CH_3_COOH); each mobile phase solvent was acidified with formic acid to a final concentration of 0.1% (*v*/*v*). Gradient: 0–5 min (5% B), 45–70 min (100% B), 80–90 min (5% B). Flow: 1.0 mL/min. Oven temperature: 25 °C. Injection volume: 10 uL for the total extract (10 mg/mL) and the butanolic fraction (5 mg/mL) [42].

High-Performance Liquid Chromatography coupled with Quadrupole-Time-of-Flight Mass Spectrometry (HPLC-QTOF/MS, Agilent Technologies, Santa Clara, CA, USA) was used for the metabolomic profiling of the hydroethanolic extract and butanolic fraction. The system consisted of an Agilent HPLC 1200 chromatograph coupled to a Q-TOF 6520 mass spectrometer, operated in positive and negative electrospray ionization (ESI) mode in separate runs. The analysis was carried out in full scan mode, covering a mass range of 50–1100 Da, with an acquisition rate of 1.0 scan/s. The source conditions of the mass spectrometer consisted of a capillary voltage of 3500 V, a nebulizer gas flow rate of 10 L/min, a pressure of 50 psi, and a source temperature of 325 °C. During all analyses, a constant mass correction was performed using two reference masses: *m*/*z* 121.0509 (C_5_H_4_N_4_, Purine) and *m*/*z* 922.0098 (C_18_H_18_O_6_N_3_P_3_F_24_, Hexakis) for the positive ionization mode and *m*/*z* 112.9856 (C_2_O_2_F_3_(NH_4_), Trifluoroacetic acid ammonium) and *m*/*z* 1033.9881 (C_18_H_18_O_6_N_3_P_3_F_24_, Hexakis) for the negative ionization mode. Zorbax extend C18 column (3.0 × 150 mm, 3.5 μm). Mobile phase: solvent A (H_2_O), solvent B (CH_3_CN), using 2 mM HCOONH_4_. Gradient: 0–2 min (5% B), 16–30 min (100% B), 35–40 min (5% B). Flow: 0.5 mL/min. Temperature: 25 °C.

The differential identification of the metabolites was carried out taking into account the precision of the mass (error < 10 ppm), the distribution of the isotopic pattern and the generation of adducts. To this end, several online public databases were consulted, including METLIN (https://metlin.scripps.edu/), KEGG (https://www.genome.jp/kegg/ (accessed on 18 August 2020)), HMDB (https://hmdb.ca/), PubChem (https://pubchem.ncbi.nlm.nih.gov/), LIPID MAPSR (https://www.lipidmaps.org/), and ChEBI (https://www.ebi.ac.uk/chebi/ (accessed on 18 August 2020)), through the CEU Mass Mediator tool (https://ceumass.eps.uspceu.es/). Confirmation of the identity of the metabolites was carried out by MS/MS analysis, using software such as MS-DIAL 4.80 (https://prime.psc.riken.jp/compms/msdial/main.html (accessed on 18 August 2020)), MS-FINDER 3.52 (https://prime.psc.riken.jp/compms/msfinder/main.html (accessed on 18 August 2020)), CFM-ID 4.0 (https://cfmid.wishartlab.com/) for simulation of in silico mass fragmentation and the GNPS platform (https://gnps.ucsd.edu/ProteoSAFe/static/gnps-splash.jsp (accessed on 18 August 2020)).

### 2.6. Statistical Analysis

A completely randomized design was used, verifying compliance with the assumptions of parametric statistics by means of normality (Shapiro-Wilks), homoscedasticity (Levene), and independence tests. An analysis of variance (ANOVA) was performed with the Tukey and Dunnett HSD post hoc mean comparison test to identify significant differences. A value of *p* < 0.05 was considered statistically significant. Data that did not present a normal distribution and homogeneity of variances were analyzed using the Friedman, Kruskal–Wallis, Dunn, and Mann–Whitney U tests. Statistical analyses were performed with GraphPad Prisma 9.5.1, Minitab 21.1.1, IBM^®^ SPSS 29.0.1.0 (171) Statistics^®^ and InfoStat 2020e [43,44].

## 3. Results and Discussion

### 3.1. Post-Treatment Clinical and Behavioral Observation

An acute toxicity analysis was performed using the Irwin/Hypocratic test and following the guidelines of the protocol for the evaluation of acute toxicity (OECD/OECD 423). During the 15 days of observation, the mice did not present adverse clinical signs associated with hydroethanolic extract or the butanolic fraction in doses administered. The reasons why no signs of toxicity were observed are possibly due to the use of CD-1 albino mice, a strain widely recognized for its consistency and robustness in toxicological studies [45]. Housing and feeding conditions were controlled, minimizing stress factors or external interference [46]. The Irwin/hippocratic test evaluated physiological and behavioral parameters, including somatomotor activity, central nervous system, reflexes, and general signs of distress, allowing even mild toxic effects to be detected. Observations made at critical points (0, 5, 15, 30, 60 min and then every day for 14 days) revealed no changes in skin, coat, eyes, mucous membranes, respiratory and circulatory systems, or in general behavior of mice [47].

### 3.2. Changes in the Body Mass of Mice

**Figure 1:**
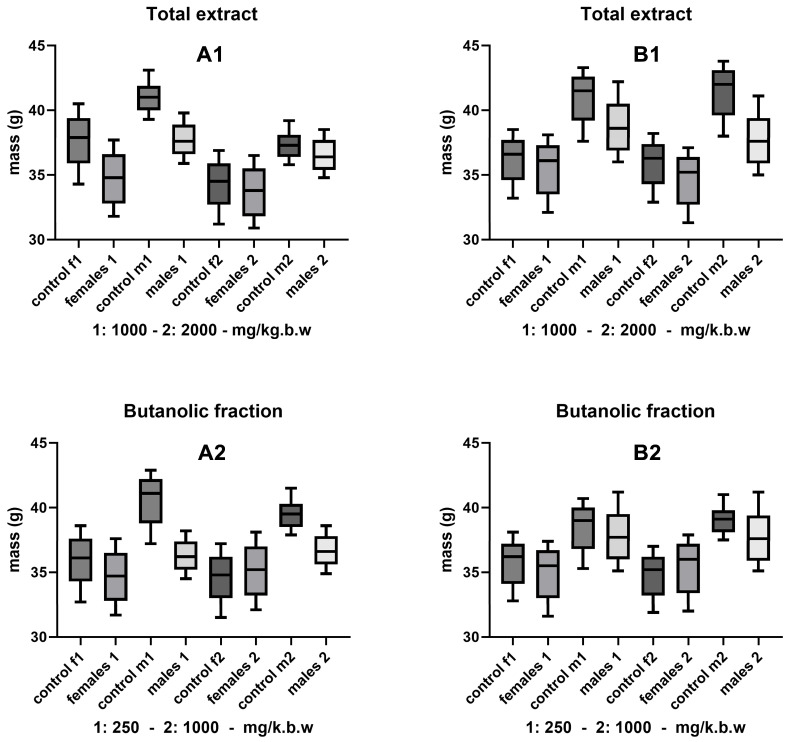
Acute single-dose toxicity. Effect of hydroethanolic extract (1000 and 2000 mg/kg bw) and butanolic fraction (250 and 1000 mg/kg bw) on body mass of mice by sex and treatment for 14 days. Oral (**A1**,**A2**) and intraperitoneal (**B1**,**B2**) routes of administration.

**Figure 2:**
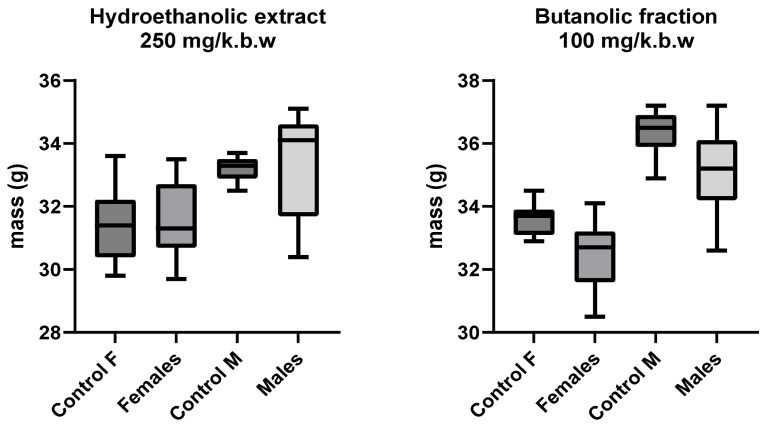
Repeated dose toxicity. Effect of hydroethanolic extract (250 mg/kg bw) and butanolic fraction (100 mg/kg bw) on body mass of mice by sex and treatment for 14 days. Oral route of administration.

Acute toxicity studies are critical to assess the safety of new bioactive compounds and their impact on model organisms. In this case, the effect of treatments on the body mass of the mice was evaluated, both in single-dose exposures (Figure 1) and in repeated doses (Figure 2), through different routes of administration (oral and intraperitoneal).

In the single-dose toxicity evaluation, no significant reductions in body mass were observed in mice treated with hydroethanolic extract at doses of 1000 and 2000 mg/kg bw, or with butanolic fraction at doses of 250 and 1000 mg/kg bw, regardless of the route of administration (oral and intraperitoneal). These results indicate favorable tolerance to the doses administered, with marginal or marginal differences between sexes in mean values. The route of administration appears to have a lesser influence on changes in body mass, suggesting that the evaluated formulations do not generate significant adverse effects in terms of weight loss, at least within the range of the doses used. This result aligns with acceptable safety standards for bioactive compounds administered experimentally in rodents [48,49].

For acute toxicity in repeated dose schedules, the results show a behavior similar to that described for single doses. Neither hydroethanolic extract (250 mg/kg bw) nor butanolic fraction (100 mg/kg bw) caused significant negative effects on the body mass of the mice during the 14 days of evaluation. Analysis by sex also did not show marked differences, which reinforces the hypothesis that treatments are well tolerated in terms of subacute toxicity. The absence of marked decreases in body mass could indicate that the compounds lack cumulative toxic effects within the exposure period, which is relevant to their safety profile. Furthermore, the differences by sex, although present, did not reach statistical significance in this experimental design [48,49].

In summary, the results obtained in both figures support the preliminary safety of the hydroethanolic extract and butanolic fraction in terms of the effects on body mass. This indicator, widely used as a surrogate marker of toxicity, confirms that the doses evaluated are tolerable and do not generate detectable adverse impacts on the measured parameters.

### 3.3. Histopathological Analysis

The present study evaluated the morphological and histopathological effects of a hydroethanolic extract and a butanolic fraction of *Piper marginatum* in a murine model, using single-dose and repeated oral and intraperitoneal regimens. The results indicate that the administration of these fractions generates predominantly mild to moderate effects, without evidence of irreversible damage to the target organs.

Vascular congestion, hepatocellular degeneration, and activated Küpffer cells were observed in the liver, findings compatible with an adaptive response to xenobiotics. These effects, although expected given the liver detoxifying role, suggest that the compounds present in the extract—particularly phenylpropanoids, chromones, and flavonoids—can induce sublethal oxidative stress under repeated conditions. Previous studies have documented the biphasic effects of *Piper* spp. metabolites, with antioxidant and pro-oxidant properties depending on dose and time of exposure [50,51].

At the renal level, signs of congestion, hypercellularity of the glomerulus, and changes suggestive of tubular cell death were observed, with no evidence of frank necrosis. These findings could be explained by the active excretion of water-soluble metabolites and their potential accumulation in glomerular and interstitial structures. Although mild, these alterations underscore the need to evaluate renal function biomarkers in subsequent studies. The lung had perivascular infiltrate, focal hemorrhages, and septal thickening, consistent with mild interstitial pneumonia. The mild local reactions observed in treated animals are likely attributable to the vehicle (propylene glycol/glycerin/water) rather than an intrinsic effect of the extract or the metabolism of bioactive terpenoids, whose pro-inflammatory action has been documented in other essential oils of the species [52].

In the heart, mild disorganization of the myocardial fibers, separation, and subcellular morphological changes were detected, without significant necrosis. Although these findings do not compromise the functional integrity of the tissue, they could be indicative of transient interference in ion channels, which is consistent with pharmacological studies on bioactive compounds of *Piper marginatum* [30]. Organs of the central nervous system (cerebrum and cerebellum) showed mild neuronal hypoxia without inflammatory infiltrate or parenchymal disorganization. These alterations, although discrete, could be related to systemic effects on perfusion rather than direct neurotoxicity, as no necrosis or gliosis was observed.

At the gastrointestinal level (stomach and intestines), the changes were minimal and compatible with mild local irritation, including epithelial desquamation and slight glandular dilation. No ulcers, necrosis, or significant structural damage was observed, suggesting good digestive tolerance of the extract at the doses evaluated, coinciding with previous studies that reported gastroprotective effects of Piper spp.-derived compounds [53]. The spleen showed mild hematopoietic activation, without evidence of inflammation or lymphoid depletion, which could reflect adaptive functional stimulation of the immune system.

Together, the histopathological data suggest that *Piper marginatum*, administered orally or intraperitoneally in single or repeated doses, produces mild to moderate impairments that do not compromise organ viability. However, the presence of recurrent findings in the liver, kidney, and lung warrants further evaluation of subchronic toxicity, genotoxicity, and biochemical biomarkers in future research. It is also recommended to establish NOAEL (no observable adverse effect level) by dose increase and complementary multiorgan analysis.

These findings are consistent with previous toxicological reports on semipolar plant fractions. For example, Wang et al. (2021) demonstrated that butanolic extracts of *Piper sarmentosum* induce mild inflammatory responses in the intestinal epithelium, which coincides with the changes observed in the lung and intestine in our study [10]. Similarly, Kulawe et al. (2020) reported that the butanolic fractions of the *Combretum molle* caused moderate alterations in the liver and kidneys of Wistar rats, suggesting that semipolar metabolites tend to concentrate compounds with toxic potential [11].

Together, these findings support our interpretation that the hepatic and renal effects in *Piper marginatum* reflect adaptive processes to xenobiotic metabolism rather than irreversible acute toxicity. Similarly, studies in other Piper species, such as *Piper nigrum* and *Piper crocatum*, have documented biphasic properties of their secondary metabolites—with antioxidant effects at low doses and pro-oxidant effects at high doses—which is consistent with the mild to moderate responses found in the liver and lung in our model [23,25].

On the other hand, regarding the absence of mortality, weight loss, or significant behavioral alterations, our results are consistent with recent studies reporting favorable safety profiles for *Piper marginatum* extracts in in vitro and preliminary in vivo trials [6,19].

Finally, we emphasize that the alterations observed in highly vascularized organs may be partially influenced by the method of sacrifice (cervical dislocation), an aspect recognized by our veterinary pathologist, who was blind to treatment and study and is a member of the Veterinary Pathology Laboratory of the Faculty of Veterinary Medicine and Zootechnics of the National University of Colombia [21,39].

It should be noted that a limitation of the present study was the use of cervical dislocation as a sacrifice method. Although this procedure is validated by international guidelines (OECD, AVMA) for its speed and absence of chemical interference in tissues, it can induce mild histopathological artifacts, such as vascular congestion and focal meningeal hemorrhages, especially in highly irrigated organs such as the lung and brain [39]. Although these alterations do not compromise the overall interpretation of the results, they should be considered when evaluating congestive or hypoxic lesions [41]. For studies focused on high-resolution neurohistological or vascular analysis, it is recommended to use deep anesthesia followed by intracardiac saline perfusion to minimize these effects [41].

Considering the presence of mild to moderate histopathological alterations in organs such as the liver, kidney, and lung, as well as the structural diversity of the metabolites identified by LC-QTOF/MS, subacute toxicity studies in complementary murine models are recommended, such as Wistar or Sprague-Dawley rats. These assays would make it possible to assess the possible accumulation of adverse effects in prolonged exposure schedules (≥28 days), to establish no observed effect levels (NOAEL) and to detect latent toxic events not visible in acute studies. It is also necessary to incorporate biochemical analyses in the blood, such as the measurement of transaminases (ALT, AST), markers of renal function (creatinine, urea), as well as hematological parameters and indicators of oxidative stress. Among the latter, the quantification of malondialdehyde (MDA) as a marker of lipid peroxidation, the determination of reduced (GSH) and oxidized (GSSG) glutathione, as well as the GSH/GSSG ratio as a key indicator of redox balance are recommended. Furthermore, the activity of antioxidant enzymes such as superoxide dismutase (SOD), catalase (CAT), and glutathione peroxidase (GPx), together with the detection of 8-hydroxy-2′-deoxyguanosine (8-OHdG) as a marker of oxidative DNA damage, would provide a deeper characterization of the functional and molecular impact of the extracts evaluated. Integration of these data will contribute to a more comprehensive assessment of the safety profile of *Piper marginatum* extracts and will allow progress toward its pharmacological validation as a potential therapeutic agent or phytopharmaceutical.

### 3.4. Preliminary Phytochemical Analysis

Preliminary phytochemical analysis performed by tube testing and Thin Layer Chromatography (TLC) allowed qualitative identification of the different classes of secondary metabolites in the total extract and butanolic fraction (Table 2). The results show the presence and absence of several key secondary metabolites, providing an initial basis for future investigations.

The presence of alkaloids was confirmed in the total extract and in the butanolic fraction by all tests performed (Ammonium reineckate, Dragendorff, Mayer, Valser and Wagner), highlighting the potential of these extracts for applications in biological activities related to antimicrobial and anticancer properties [54,55].

The Kedde and Vanillin/H_3_PO_4_/Kedde assays did not detect the presence of cardiotonic glycosides in the total extract or the butanolic moiety. However, these compounds were confirmed in the positive control. This could indicate that although cardiotonic glycosides are absent in the evaluated samples, the methods used are effective for their detection in other matrices [56]. Coumarins were detected only in the total extract and the butanolic fraction using the Vanillin/H_3_PO_4_/FeCl_3_/HCl assay. This suggests a moderate distribution of specific coumarins using the TLC method. These metabolites are associated with antioxidant and photoprotective properties [57].

Flavonoids were present in both the total extract and the butanolic fraction, confirmed by NP-PEG and Shinoda tests. This highlights the antioxidant and anti-inflammatory potential of these matrices, given the well-known bioactive profile of flavonoids [58]. Differences in the distribution of naphtho- and/or anthraquinones were observed. Although the total extract showed a positive response in the Borntränger-Krauss and KOH/EtOH assays, the butanolic fraction did not present positive results. This suggests that these compounds could be present in other fractions of lower polarity, such as the dichloromethane fraction, or in the aqueous fraction if they are hydroxylated. Their relevance lies in their traditional use as antibacterial, antifungal, antimalarial, and anticancer agents, among others [59].

Tannins and proanthocyanidins were detected in the total extract and butanolic fraction by tests such as gelatin salt, potassium ferricyanide, and BuOH/HCl. Their presence highlights the possible application of these extracts in antimicrobial activities and as antioxidant agents [60,61]. Saponins were absent in both matrices, according to foaming, hemolysis, and anisaldehyde/H_2_SO_4_ tests. Although positive control confirmed the efficacy of the methods used, the absence of saponins limits applications related to surfactant or modulating activities of the immune system [62].

Steroids and/or triterpenes were detected in the total extract by the Liebermann-Burchard and Vanillin tests, but were absent in the butanolic fraction. This indicates an affinity for less polar fractions, such as those obtained with organic solvents [63]. Like steroids, terpene lactones were detected in the total extract by tests such as ferric hydroxamate and Vanillin/H_3_PO_4_/FeCl_3_/HCl. Their presence highlights the potential of these matrices in antimicrobial and antiparasitic activities [64].

### 3.5. Chromatographic Profiling by HPLC/PDA and LC-QTOF/MS

**Figure 3:**
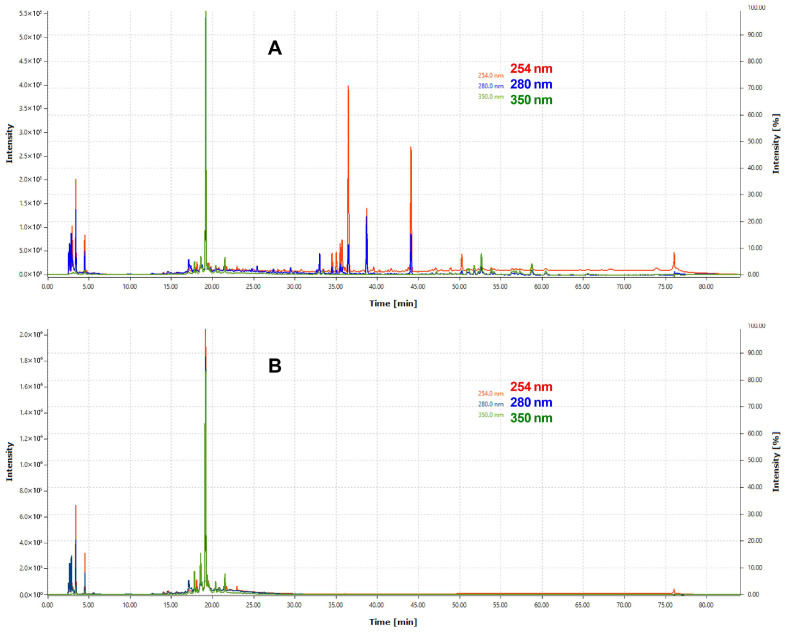
HPLC/PDA profiling of the total extract (**A**) and the butanolic fraction (**B**) of *Piper marginatum*, at three wavelengths, 254, 280 and 350 nm. Openchrom 1.5.0 Data Processing Software.

Analysis of the chromatographic profiles obtained by HPLC/PDA (Figure 3) allowed us to evaluate the chemical complexity and distribution of secondary metabolites in the total hydroethanolic extract and the butanolic fraction of *Piper marginatum*. This analysis was based on the absorption at different wavelengths (254, 280 and 350 nm).

In the total hydroethanolic extract (A), the chromatogram reveals a greater chemical complexity, with multiple peaks distributed throughout the retention time (0–80 min). At 254 nm, a high density of peaks is observed in the initial range (0–20 min) and in the middle range (30–50 min), indicating the presence of aromatic compounds and conjugated bonds. At 280 nm, peaks between 35 and 40 min stand out, possibly associated with phenolic compounds or flavonoids. At 350 nm, fewer peaks and lower intensity are detected, suggesting that absorbing compounds at this wavelength, such as flavonoids with extensive conjugated systems, are less abundant. Some dominant peaks exhibit absorption at all wavelengths, indicating the presence of polyfunctional compounds with multiple chromophore groups [65,66,67].

In contrast, the chromatogram of the butanolic fraction (B) is less complex, with the main peaks concentrated between 15 and 25 min. A dominant peak around 20 min, detected at 254 and 280 nm, suggests the presence of a major secondary metabolite with high affinity for butanol. At 350 nm, the signal is less prominent, reflecting a lower abundance of compounds with highly conjugated structures. The reduction in chromatographic complexity with respect to the total extract indicates the enrichment of specific metabolites in this fraction [65,66,67].

The absorption profile at different wavelengths reinforces these observations (Table 3). The total hydroethanolic extract exhibits a higher number of peaks at all wavelengths analyzed (191, 180 and 108 peaks at 254, 280 and 350 nm, respectively), indicating a high chemical diversity. In particular, the 191 peaks at 254 nm suggest a wide variety of compounds with absorbent chromophores, such as simple and conjugated aromatic systems. However, the butanolic fraction shows a lower number of peaks (77, 77 and 47, respectively), reflecting the effect of fractionation on the concentration of certain secondary metabolites and the elimination of less polar compounds or compounds with low affinity for butanol.

Analysis of the total area complements this characterization. The hydroethanolic extract has larger areas at all wavelengths, with outstanding values at 254 nm (8.30 × 10^9^) and 280 nm (8.10 × 10^9^), indicating a higher concentration of compounds with moderate aromatic or conjugate systems. On the other hand, the butanolic fraction has lower areas (5.10 × 10^9^ at 254 nm and 1.40 × 10^9^ at 350 nm), reflecting a lower chemical complexity and concentration of specific metabolites.

Butanolic fractionation reduces the complexity of the chemical profile of the total hydroethanolic extract by concentrating specific metabolites and removing compounds with lower affinity for the solvent. The larger areas at 254 and 280 nm in both cases suggest that the predominant compounds are simple aromatics or possess π-conjugated systems. However, the low intensity at 350 nm indicates a lower presence of highly conjugated compounds, such as complex flavonoids. These findings, along with the spectral profile of the majority peak, support the hypothesis that the predominant compounds in *Piper marginatum* include simple phenols, flavonoids, and lignans with moderate conjugation.

In conclusion, HPLC/PDA chromatographic analysis demonstrates that the total hydroethanolic extract has a higher chemical diversity, while butanolic fractionation allows a specific subset of secondary metabolites to be selected and concentrated. The 254 nm wavelength stands out for identifying the highest number of peaks and offering high analytical sensitivity in this type of sample. These results are relevant for the identification of bioactive compounds, as they suggest that the metabolites present in the butanolic fraction could be related to the biological activities reported for *Piper marginatum* [14,15,18,32].

The tentative identification of the compounds in the samples was performed using LC-QTOF/MS. The following compounds were identified in the hydroethanolic extract: ethylenethiourea, lupanyl acid, eriobofuran, pinocembrin, 9-methoxyellipticine, 4-hydroxynornantenine, 5-deoxystrigol, dehydroevodiamine, niazimicin, sinapoyltyramine, asperidine C, C17-sphingosine-1-phosphate, N-methyl tetramethoxy chrysoaranoic acid, desglucocoroloside, D-glucosyldihydrosphingosine, spinatoside, prenylflavan-7-O-glucoside, phosphatidylethanolamine methoxyalkyl (PE-OCH_3_ C21:0) and carboxymethylated phosphatidylperoxidiinylglycerol (Table 4).

One of the compounds identified was ethylenethiourea, a sulfur heterocycle derived from imidazolidine, with a possible synthetic origin, known for its use as a vulcanizing agent and for its toxic effects in carcinogenesis and teratogenicity studies in animal models [68,69]. Its presence in natural extracts could indicate contamination or secondary biotransformation. Lupanyl acid, a nitrogenous lupanoid derivative, was identified as a triterpene with a polycyclic structure. These types of compounds have been associated with anti-inflammatory and antitumor effects, probably due to their interaction with nuclear receptors and modulation of signaling pathways such as NF-κB [72]. The compound eriobofuran, a derivative of dibenzofuran, has previously been described in plant extracts with insecticidal activity and antioxidant potential, possibly mediated by inhibition of free radicals [76]. Pinocembrin, a widely distributed flavanonol in medicinal plants, possesses anti-inflammatory, antimicrobial, and neuroprotective properties. It has been reported to exert its effects by inhibiting the COX-2, iNOS, and modulating the MAPK and NF-κB pathways [80].

Among the alkaloids detected, 9-methoxyellipticine, a pyridoid[4,3-b]carbazolic alkaloid with documented antitumor activity due to its ability to intercalate in DNA and generate reactive oxygen species (ROS), inducing apoptosis in cancer cells [82]. Likewise, 4-hydroxynornantenine, an indole alkaloid, has an unusual pentacyclic architecture; although its specific bioactivity is not fully characterized, structurally related compounds show spasmolytic activity and effects on the central nervous system [87]. 5-deoxystrigol strigolactonide was detected as a possible regulator of plant growth and mediator of rhizospheric interactions. These compounds have also been proposed as candidates for the treatment of diseases due to their modulation of Wnt-like signaling pathways [91]. Dehydroevodiamine, a quinazolinoid alkaloid, has been studied for its effects on cholinergic neurotransmission and its neuroprotective potential, which could have applications in diseases such as Alzheimer’s [93]. In the same vein, niazimicin, a phenolic thiocarbamate, is known for its anticancer activity and the ability to inhibit TPA-induced carcinogenesis, possibly for its effect on cell proliferation and inhibition of xenobiotic metabolizing enzymes [97].

The phenylpropanoid sinapoyltyramine exhibits antioxidant properties and has been associated with lignification processes in plants. Its biological action could be related to inhibition of lipid peroxidation and modulation of phenolic metabolism [102]. Within the bicyclic alkaloids, asperidine C showed an unusual azabicyclic structure and has been associated with antimicrobial and antiprotozoal activities [103]. Among bioactive lipids, two sphingolipids were identified: C17 sphingosine-1-phosphate, involved in the regulation of apoptosis and cell migration through the S1P1-S1P5 receptors [109], and D-glucosyldihydrosphingosine, a structural glycosphingolipid present in cell membranes, with functions in signaling and adhesion [118]. The alkaloid N-methyl tetramethoxychrysoaranoic acid contains a benzo[c]phenanthridine nucleus and has been associated with cytotoxic and antibacterial activity, particularly in Zanthoxylum and Chelidonium species [110]. Desglucocoroloside, a cardenolide, represents a steroidal glycoside related to positive inotropic effects on the myocardium by inhibiting Na^+^/K^+^-ATPase, which justifies its cardiotoxic potential if its dose is not controlled [115].

In the flavonoid group, spinatoside was found, a flavone glycoside that includes glucuronic acid and methoxy groups, which could improve its bioavailability and antioxidant activity [121]. Furthermore, prenylflavan-7-O-glucoside combines prenylation and glycosylation, two modifications that improve permeability and biological activity, especially in terms of antifungal and antiproliferative activity [124]. Finally, complex lipids such as phosphatidylethanolamine methoxyalkyl (PE-OCH_3_ C21:0) and carboxymethylated phosphatidylperoxidiinoyinoyglycerol were detected, compounds that could participate in cell signaling, inflammation, and oxidative stress processes, although their specific role in plant extracts requires further investigation [128,131].

Together, these findings suggest that *Piper marginatum* possesses a rich and diverse phytochemical matrix, with multiple compounds of potential pharmacological relevance. Identification of metabolites with anticancer, antioxidant, anti-inflammatory, and cardiotonic properties supports the ethnopharmacological interest of this species and justifies its study as a source of phytomedicines or as a model for the development of new bioactive ingredients.

Analysis of the butanolic fraction of the hydroethanolic extract of *Piper marginatum* revealed a high diversity of secondary metabolites, with a predominance of phenylpropanoids, lactones, alkaloids, benzofurans, sphingolipids and azacycloaliphatic compounds (Table 5). The following compounds were identified: piperonylic acid, lastar A, coniferyl aldehyde, 3-veratril-1-propanol, kakuol, safrolglycol, loliolide, syringylacetone, pogostone, acoramone, isoacoramone, aspidinol, senkyunolide I, lupanyl acid, dihydrobaicalein, C16-sphinganine, coriandrone B, N-isopalmitoylpyrrolid, feruloyl tyramine, phytosphingosine, galactosyl-DNJ, pipercitine, and pheophorbide A (Table 5).

Among the most representative compounds was piperonylic acid, an acid derivative of benzodioxol structurally related to safrole. This compound has been described as an inhibitor of oxidative enzymes and a modulator of pathways associated with cellular detoxification [133]. Similarly, the alkaloid Lastar A (2,2,6,6-tetramethyl-4-piperidinol) is a cyclic amine with a piperidine structure that has shown potential antioxidant activity and has been used as a stabilizer in polymeric systems, although its toxicity in biological systems still requires a more detailed evaluation [137]. Phenylpropanoids detected include coniferyl aldehyde, a key compound in lignin biosynthesis, with reports of antimicrobial and anti-inflammatory activity [140], and 3-veratril-1-propanol, an aromatic alcohol that could act as a precursor to lignans with neuroprotective effects [142]. Other structurally related compounds, such as acoramone, isoacoramone, aspidinol, and syringylacetone, share a substituted propanon nucleus and have been associated with antiseptic and anti-inflammatory effects, mainly in ethnopharmacological studies of Acorus and Syringa species [172].

In the lactone class, senkyunolide I, previously reported in *Ligusticum chuanxiong*, was identified with vasodilatory and antiplatelet effects by inhibiting intracellular Ca^2+^-dependent pathways [175]. Loliolide, a lactonic benzofuranone with antioxidant and anti-aging properties, which is present in marine and terrestrial plant species, was also detected [155]. Kakuol and safrolglycol are derivatives of 1,3-benzodioxol and have been reported as bioactive compounds with moderate antifungal and anxiolytic activity, although hepatotoxicity associated with safrole metabolites has been documented in murine models [152]. The alkaloid lupanyl acid, also present in the total extract, reaffirms its persistence in fewer polar fractions and suggests a lipophilic affinity compatible with its dense triterpenoid structure. On the other hand, the flavonoid dihydrobaicalein, a trihydroxylated flavanonide, shows antioxidant and cytoprotective potential against oxidative stress in studies with hepatocytes [179]. The fraction also presented nitrogenous lipids such as C16 sphinganine and phytosphingosine, sphingoid bases with essential functions in apoptotic signaling and in the structural organization of cell membranes. These lipids have been proposed as modulators of the immune response and as therapeutic targets in dermatological and neurodegenerative diseases [186,201].

Another compound of interest was feruloyl tyramine, a hydroxycinamamide with antioxidant, monoamine oxidase (MAO) inhibitory properties, and antidepressant potential [195]. Galactosyl-DNJ was also identified, an iminosaccharide derived from nojirimycin that acts as a glucosidase inhibitor, with therapeutic applications in lysosomal diseases and as an antidiabetic due to its hypoglycemic action [204. It should also be noted that the presence of pipercitine, a long-chain aliphatic azacycloalkane with an amide function and a terminal piperidin. These types of compounds have been associated with cytotoxic mechanisms due to membrane disruption and alteration of lipid fluidity [209]. Finally, pheophorbide A, a chlorophyll derivative type tetrapyrrole, with recognized photosensitizing and cytotoxic action, was detected, and pheophorbide A was detected experimentally, which was used in photodynamic therapy against skin and gastrointestinal cancers [213].

The structural and functional richness of the metabolites present in the butanolic fraction indicates a high degree of chemical specificity, with an eye on semipolar and lipophilic compounds with relevant pharmacological activities. This fraction complements the profile of the total extract and suggests that *Piper marginatum* contains phytochemicals with multiple mechanisms of action, from antioxidant and neuroprotective effects to potential oncological and antidiabetic applications.

Characterization of the chemical profile of the hydroethanolic extract and its butanolic fraction of *Piper marginatum* was performed using high-performance liquid chromatography coupled to mass spectrometry with a quadrupole time-of-flight analyzer (HPLC-QTOF/MS), one of the most widely used platforms in studies of untargeted metabolomics and exploratory phytochemistry. This technique provides high mass resolution and high accuracy, allowing for the inference of exact molecular formulas and the tentative identification of compounds in complex samples [214,215]. Among the strengths highlighted, HPLC-QTOF/MS allows for a broad chemical coverage, detecting compounds with a range of masses, from primary metabolites such as amino acids to lipophilic secondary metabolites such as alkaloids, lipids, flavonoids and terpenoids [216]. In addition, the ability to acquire MS/MS fragmentation spectra in real time or through DDA (data-dependent acquisition) facilitates comparative structural elucidation against libraries such as MassBank, METLIN, GNPS or HMDB [217,218].

The ability to assign molecular identities without requiring pure standards is another significant operational advantage, especially in the research of understudied natural products or plant extracts [219]. This aspect was essential in our study for the annotation of compounds such as pinocembrin, synapoyl-tyramine, or galactosyl-DNJ, which were identified by exact mass matching, fragmentation pattern, and molecular formula. However, there are important methodological limitations. Non-standard identification is classified according to the Metabolomics Standards Initiative (MSI) into confidence levels ranging from level 1 (confirmed identification) to level 4 (unknown mass) [220]. In our study, most of the annotations reached a level 2 (probable structure) or level 3 (chemical class), which implies a margin of uncertainty that must be recognized in biological interpretation. Another major challenge is the formation of multiple ionic adducts (M+H^+^, M+Na^+^, M+NH_4_^+^, H_2_O, etc.), which can hinder the correct assignment of the precursor ion and lead to redundant or misinterpreted peaks [221]. This is aggravated in complex plant matrices, such as crude extracts, where the presence of salts, polar compounds, and coeluents can induce ion suppression and signal loss [222]. In addition, reliance on public spectral libraries limits the annotation of new or poorly characterized compounds. For example, several compounds tentatively identified in our analysis, such as pipercetin or phosphatidylperoxidinoylglycerol, did not have MS/MS spectra recorded, preventing confirmation beyond level 3. This limitation underscores the need for complementary techniques such as NMR for definitive structural validation [223,224].

In summary, the use of HPLC-QTOF/MS provides a powerful and sensitive tool for the analysis of plant extracts such as those of *Piper marginatum*, allowing the discovery of bioactive metabolites with robust preliminary characterization. However, its results must be interpreted with analytical criteria and complemented, in the future, with structural spectroscopy to achieve unequivocal identifications and well-founded pharmacological applications.

### 3.6. Strengths and Weaknesses of This Study

This study presents several strengths, including the use of the OECD 423 guideline, a balanced experimental design with both sexes, and a comprehensive evaluation that combined clinical, histopathological, and advanced phytochemical analyses (HPLC-PDA and LC-QTOF/MS), supported by blinded histological evaluation. Among the limitations, serum biomarkers were not evaluated, most metabolite identifications correspond to MSI levels 2–3, requiring NMR confirmation, and the use of cervical dislocation as the euthanasia method, although internationally validated, can introduce mild histological artifacts in highly vascularized organs. Furthermore, LD_50_ values are not applicable to complex multicomponent plant extracts, as this parameter is restricted to single defined compounds; therefore, a more appropriate approach is to establish the level of no observed adverse effects (NOAEL) through subacute or subchronic studies, which would provide realistic and comparable safety margins in accordance with international regulatory guidelines. Despite these limitations, the methodological strengths outweigh potential weaknesses, and the results provide a solid basis for future studies of the mechanistic and subchronic toxicity of *Piper marginatum* extracts.

## 4. Conclusions

The present study shows that both the hydroethanolic extract and the butanolic fraction of *Piper marginatum* Jacq. present a favorable acute toxicity profile in CD-1 mice, without significant alterations in behavior, body weight, or the histology of vital organs at the doses evaluated. The histopathological alterations observed were mild to moderate and did not compromise the functionality of the target organs, suggesting a physiological adaptive response rather than a direct toxic effect. The phytochemical composition revealed by qualitative analyses and LC-QTOF/MS confirms the presence of bioactive metabolites of pharmacological interest, including flavonoids, alkaloids, complex lipids and phenolic compounds. The absence of relevant acute toxic effects supports the advancement of the preclinical development of this plant species. In general, this research contributes significantly to the toxicological and phytochemical knowledge of *Piper marginatum* and strengthens its projection as a safe and potentially applicable phytomedicine in therapeutic contexts.

## Figures and Tables

**Table 1:** Description of acute toxicity in mice.

Dose	Route of Administration	Substance	mg/kg bw	Number of Animals *	Age (Weeks)
Unique	Oral e intraperitoneal	Total extract	2000	8	8–9
			1000	8	
		Butanolic fraction	1000	8	
			250	8	
Repeated	Oral	Total extract	250	8	
		Butanolic fraction	100	8	
Control	Oral and intraperitoneal	Vehicle	-	8	

* Eight animals (4 males and 4 females) were used for a single dose evaluation, and eight animals (4 females and 4 males) for repeated dose evaluation.

**Table 2:** Preliminary phytochemical analysis using qualitative wet chemistry tests and Thin-Layer Chromatography.

Metabolite Class (Test) (*, **)	Total Extract	Butanolic Fraction	Control	
			A (−)	B (+)
Alkaloids (ammonium reineckate) *	+	+	−	+
Alkaloids (Dragendorff) *	+	+	−	+
Alkaloids (Mayer) *	+	+	−	+
Alkaloids (Valser) *	+	+	−	+
Alkaloids (Wagner) *	+	+		
Cardiotonic glycosides (Kedde) *	−	−	−	+
Cardiotonic glycosides (Vanillin/H_3_PO_4_/Kedde) **	−	−	−	+
Coumarins (fluorescence) *	−	−	−	+
Coumarins (Vainillin/H_3_PO_4_/FeCl_3_/HCl) **	+	+	−	+
Flavonoids (NP-PEG) **	+	+	−	+
Flavonoids (Shinoda) *	+	+	−	+
Leucoanthocyanin (Rosenhein) *	−	−	−	+
Naphtho- and/or anthraquinones (Borntränger-Krauss) *	+	−	−	+
Naphtho- and/or anthraquinones (KOH/EtOH) **	+	−	−	+
Phenols (ferric chloride) *	+	+		
Proanthocyanidins (BuOH/HCl) *	+	+	−	+
Saponins (Anisaldheido/H_2_SO_4_) **	−	−	−	+
Saponins (foam) *	−	−	−	+
Saponins (Hemolysis) *	−	−	−	+
Steroids and/or triterpenes (Liebermann-Burchard) *	+	−	−	+
Steroids and/or triterpenes (Vanillin) **	+	−		
Tannins (gelatin, salt and ferric chloride) *	+	+	−	+
Tannins (potassium ferrocyanide and ferric chloride) **	+	+	−	+
Terpenic lactones (ferric hydroxamate) *	+	−	−	+
Terpenic lactones (Vainillin/H_3_PO_4_/FeCl_3_/HCl) **	+	−	−	+

* Tube test. ** Test by thin-layer chromatography. Hydroethanolic extract (7:3) and butanolic fraction of *Piper marginatum*. ‘+’ = Presence. ‘−’ = absence. A = Negative control (−): H_3_O^+^ for alkaloids. Hexane for steroids. Ethanol: water (1:7) for flavonoids, saponins, tannins. Toluene for naphtho and/or anthraquinones. Chloroform for steroids, terpenic lactones, cardiotonic glycosides, and coumarins. B = positive control (+): Alkaloids extracted from Berberine and *Brugmansia arborea*. Lupeol for steroids and/or triterpenes. Rutin and/or quercetin for flavonoids. Anthrones and extracts of *Rheum rhabarbarum* and *Cassia angustifolia* for naphtho and/or anthraquinones. *Cecropia metensis* extract for tannins. Extract of Passiflora edulis for saponins. Digitoxin for cardiotonics. Umbelliferone for coumarins.

**Table 3:** UV-vis absorption profile for total extract and butanolic fraction of *Piper marginatum* at different wavelengths.

Extract/Fraction	Parameters	254 nm	280 nm	350 nm
Hydroethanolic extract	Number of peaks	191	180	108
	Total Area *	8.30 × 10^9^	8.10 × 10^9^	2.40 × 10^9^
Butanolic fraction	Number of peaks	77	77	47
	Total area *	5.10 × 10^9^	4.80 × 10^9^	1.40 × 10^9^

* Arbitrary units generated by software.

**Table 4:** Hydroethanolic extract of *Piper marginatum*—Tentative identification of secondary metabolites by LC-QTOF/MS.

t_R_ min	*m*/*z*			Adduct	Formula	Tentative Identification			Metabolite Class	References
	Exp	Theoric	Error ppm			Common Name	IUPAC Name	ID Level		
20.12	125.0149	102.0252	5	M+Na	C_3_H_6_N_2_S	Ethylenethiourea	Imidazolidine-2-thione	3	Sulphur and heterocyclic compound (imidazole)	[68,69,70,71]
15.19	235.1810	234.1732	1	M+H	C_14_H_22_N_2_O	Lupanyl acid	3-(7,11-diazatricyclo[7.3.1.0~2,7~]tridecan-10-yl)propanoic acid	3	Triterpenoid derivative (lupanoid)	[72,73,74]
20.12	243.0683	244.0736	8	M−H	C_14_H_12_O_4_	Eriobofuran	2,4-dimethoxydibenzofuran-3-ol	3	Dibenzofuran derivative (furanopheno)	[75,76,77]
18.16	255.0682	256.0736	7	M−H	C_15_H_12_O_4_	Pinocembrin	(2S)-5,7-dihydroxy-2-phenyl-2,3-dihydrochromen-4-one	3	Flavonoid (flavanonol)	[78,79,80,81]
23.63	277.1339	276.1263	1	M+H	C_18_H_16_N_2_O	9-Methoxyellipticine	9-methoxy-5,11-dimethyl-6H-pyrido[4,3-b]carbazole	3	Alkaloid (pyrido carbazole)	[82,83,84,85]
18.06	324.1265	341.1263	9	M+H−H_2_O	C_19_H_19_NO_5_	4-Hydroxynornantenine	18,19-dimethoxy-5,7-dioxa-13-azapentacyclo[10.7.1.0~2,10~.0~4,8~.0~16,20~]icosa-1(20),2,4(8),9,16,18-hexaen-15-ol	3	Alkaloid indólico	[86,87,88]
18.16	329.1425	330.1467	9	M−H	C_19_H_22_O_5_	5-Deoxystrigol	(3E,3aR,8bS)-8,8-dimethyl-3-[[(2R)-4-methyl-5-oxo-2H-furan-2-yl]oxymethylidene]-3a,4,5,6,7,8b-hexahydroindeno[1,2-b]furan-2-one	3	Strigolactone and indenofuran	[89,90,91]
22.46	336.0901	301.1215	3	M+Cl	C_19_H_15_N_3_O	Dehydroevodiamine	21-methyl-3,13,21-triazapentacyclo[11.8.0.0~2,10~.0~4,9~.0~15,20~]henicosa-1,3,5,7,9,15,17,19-octaen-14-one	3	Alkaloid (quinazoline)	[92,93,94,95]
21.83	340.1218	357.1246	1	M+H−H_2_O	C_16_H_23_NO_6_S	Niazimicin	O-ethyl N-[[4-(3,4,5-trihydroxy-6-methyloxan-2-yl)oxyphenyl]methyl]carbamothioate	3	Sulphur compound	[96,97,98]
17.73	342.1371	343.1420	7	M−H	C_19_H_21_NO_5_	Sinapoyltyramine	(E)-3-(4-hydroxy-3,5-dimethoxyphenyl)-N-[2-(4-hydroxyphenyl)ethyl]prop-2-enamide	3	Phenylpropanoid derivative	[99,100,101,102]
21.68	366.2182	331.2511	7	M+Cl	C_21_H_33_NO_2_	Asperidine C	(2R,4S,5R,6S)-2-nonyl-6-phenyl-7-oxa-1-azabicyclo[3.2.1]octan-4-ol	3	Alkaloid (azabicyclic)	[103,104,105]
19.01	400.2030	365.2331	1	M+Cl	C_17_H_36_NO_5_P	C17 Sphingosine-1-phosphate	[(E,2S,3R)-2-amino-3-hydroxyheptadec-4-enyl] dihydrogen phosphate	3	Sphingolipid	[106,107,108,109]
23.89	422.1639	423.1682	7	M−H	C_24_H_25_NO_6_	N-methyl tetramethoxy chrysoaranoic acid *	2-[(6S)-2,3,7,8-tetramethoxy-5-methyl-6H-benzo[c]phenanthridin-6-yl]acetic acid	3	Alkaloid (benzo[c]phenanthridine)	[110,111,112]
34.69	485.2924	504.3087	4	M−H−H_2_O	C_29_H_44_O_7_	Desglucocoroloside	3-[3-(4,5-dihydroxy-6-methyloxan-2-yl)oxy-14-hydroxy-10,13-dimethyl-1,2,3,4,5,6,7,8,9,11,12,15,16,17-tetradecahydrocyclopenta[a]phenanthren-17-yl]-2H-furan-5-one	3	Cardenolide glycoside	[113,114,115]
34.67	502.3178	463.3509	7	M+K	C_24_H_49_NO_7_	D-glucosyldihydrosphingosine	(2R,3R,4S,5S,6R)-2-(2-amino-3-hydroxyoctadecoxy)-6-(hydroxymethyl)oxane-3,4,5-triol	3	Glycosphingolipid	[116,117,118,119]
28.52	540.1365	522.1010	3	M+NH_4_	C_23_H_22_O_14_	Spinatoside	(2S,3S,4S,5R,6S)-6-[4-(5,7-dihydroxy-3,6-dimethoxy-4-oxochromen-2-yl)-2-hydroxyphenoxy]-3,4,5-trihydroxyoxane-2-carboxylic acid	3	Flavonoid glycoside (O-glycosylated type with glucuronic acid)	[120,121,122]
22.85	557.1967	534.2101	5	M+Na	C_27_H_34_O_11_	Prenylflavan-7-O-glucosideo **	(2S,4S,5S)-2-[[(2R,3S,4S)-3,4-dihydroxy-2-(4-hydroxyphenyl)-5-methoxy-8-(3-methylbut-2-enyl)-3,4-dihydro-2H-chromen-7-yl]oxy]-6-(hydroxymethyl)oxane-3,4,5-triol	3	Flavandiol prenylated glucoside	[123,124,125,126,127]
34.70	584.3969	539.3951	7	M+HCOOH−H	C_27_H_58_NO_7_P	Methoxyalkyalkylated phosphatidylethanolamine (PE-OCH_3_ C21:0) ***	2-aminoethyl [(2R)-2-hydroxy-3-(2-methoxyhenicosoxy)propyl] hydrogen phosphate	3	Ether-type phosphatidylethanolamine	[128,129]
34.69	986.6010	963.6201	9	M+Na	C_53_H_90_NO_12_P	Carboxymethylated phosphatidylperoxydinoxyenoylglycerol ****	2-[2-[[(2R)-2,3-bis(docosa-9,11-diynylperoxy)propoxy]-hydroxyphosphoryl]oxyethyl-(carboxymethyl)amino]acetic acid	3	Phospholipid	[130,131]
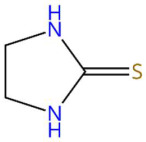						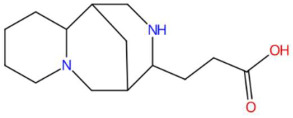		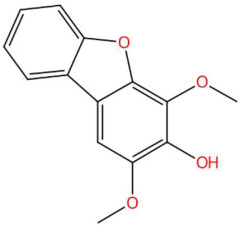		
Ethylenethiourea (t_R_ 20.12 min; C_3_H_6_N_2_S) 102.0252 Da						Lupanyl acid (t_R_ 15.19 min; C_14_H_22_N_2_O) 234.1732 Da		Eriobofuran (t_R_ 20.12 min; C_14_H_12_O_4_) 244.0736 Da		
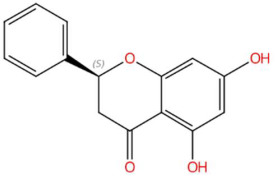						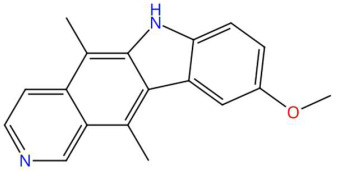		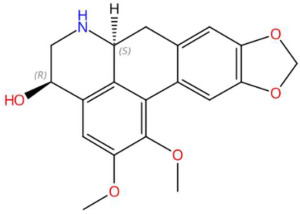		
(-)-Pinocembrin (t_R_ 18.16 min; C_15_H_12_O_4_) 256.0736 Da						9-Methoxyellipticine (t_R_ 23.63 min; C_18_H_16_N_2_O) 276.1263 Da		4-Hydroxynornantenine (t_R_ 18.06 min; C_19_H_19_NO_5_) 341.1263 Da		
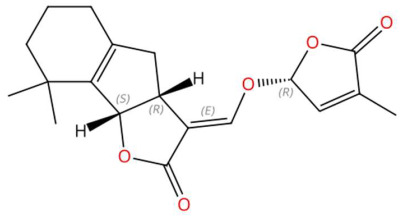						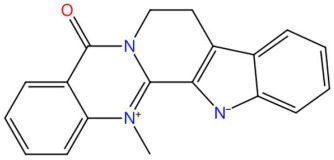		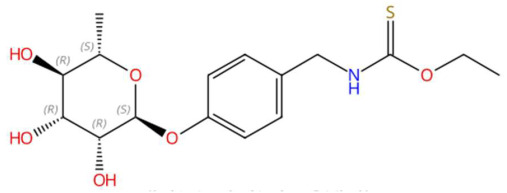		
5-Deoxystrigol (t_R_ 18.16 min; C_19_H_22_O_5_) 330.1467 Da						Dehydroevodiamine (t_R_ 22.46 min; C_19_H_15_N_3_O) 301.1215 Da		Niazimicin (t_R_ 21.83 min; C_16_H_23_NO_6_S) 357.1246 Da		
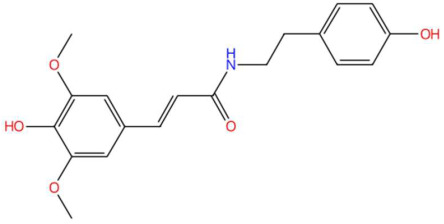						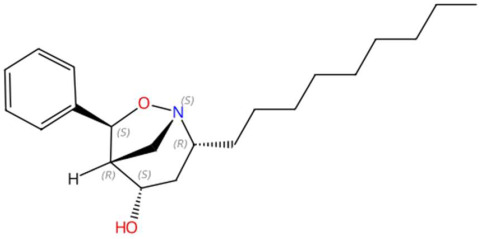		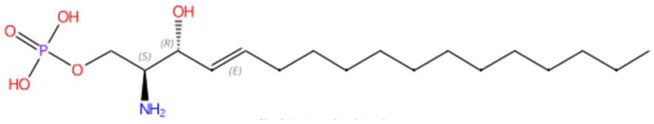		
Sinapoyltyramine (t_R_ 17.73 min; C_19_H_21_NO_5_) 343.1420 Da						Asperidine C (t_R_ C 21.68 min; C_21_H_33_NO_2_) 331.2511 Da		C17 Sphingosine-1-phosphate (t_R_ 19.01 min; C_17_H_36_NO_5_P) 365.2331 Da		
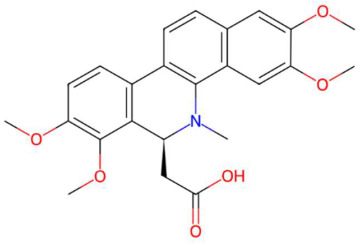						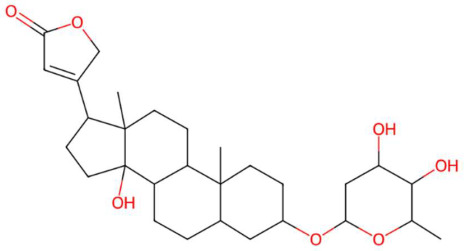		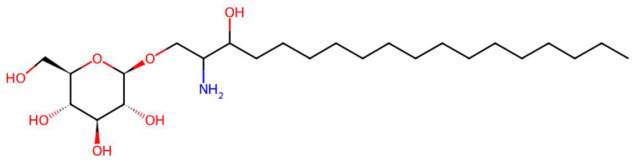		
N-methyl tetramethoxy chrysoaranoic acid (t_R_ 23.89 min; C_24_H_25_NO_6_)—423.1682 Da						Desglucocoroloside (t_R_ 34.69 min; C_29_H_44_O_7_) 504.3087 Da		D-glucosyldihydrosphingosine (t_R_ 34.67 min; C_24_H_49_NO_7_) 463.3509 Da		
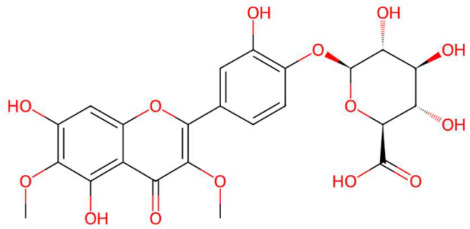						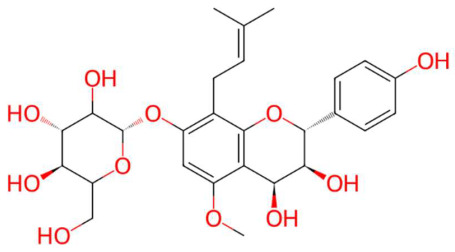		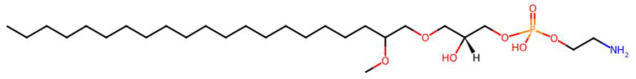		
Spinatoside (t_R_ 28.52 min; C_23_H_22_O_14_) 522.1010 Da						Prenylflavan-7-O-glucosideo (t_R_ 22.85 min; C_27_H_34_O_11_) 534.2101 Da		Methoxyalkyalkylated phosphatidylethanolamine (t_R_ 34.70 min; C_27_H_58_NO_7_P)—539.3951 Da		
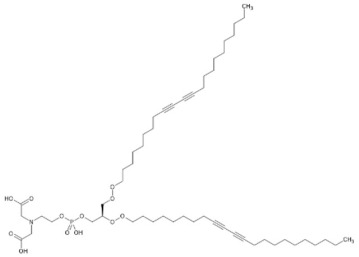										
Carboxymethylated phosphatidylperoxydinoxyenoylglycerol (t_R_ 34.69 min; C_53_H_90_NO)—963.6201 Da										

* The compound identified as N-Methyl-2,3,7,8-tetramethoxy-5,6-dihydrobenzo[c]phenanthridine-6-ethanoic acid (PubChem CID: 443717) is designated in this study as N-methyltetramethoxyrisoaranoic acid, an abbreviated and systematically descriptive form based on its phenanthrenoisoquinoline nucleus. This common name was selected to facilitate chemical communication in the phytochemical context, highlighting both the carboxylic functionality and the methoxy and nitrogen substitutions that characterize its structure. ** The compound identified as (2S,4S,5S)-2-[[(2R,3S,4S)-3,4-dihydroxy-2-(4-hydroxyphenyl)-5-methoxy-8-(3-methylbut-2-enyl)-3,4-dihydro-2H-chromen-7-yl]oxy]-6-(hydroxymethyl)oxane-3,4,5-triol (PubChem CID: 44257161) has been designated in this study with the common name “prenilflavan-7-O-glucoside”, in order to facilitate its reference within the text and figures. This name summarizes the most relevant structural characteristics of the metabolite: the flavonoid nucleus, prenylation at the C-8 position, and glycosylation at C-7. Its use responds to criteria of clarity, taxonomic simplification and coherence with the nomenclature used in phytochemical studies of prenylated and glycosylated flavonoids of natural origin. *** The compound identified as 2-aminoethyl [(2R)-2-hydroxy-3-(2-methoxyhenicosoxy)propyl] hydrogen phosphate (PubChem CID: 137323927) was functionally classified as methoxyalkyl phosphatidylethanolamine (PE-OCH_3_ C21:0). This common name highlights both the nature of the polehead (phosphorylated ethanolamine) and the presence of a long C21 alkyl chain with a methoxy substitution, facilitating its interpretation within the structural analysis of lipids in metabolomic and phytochemical studies. **** To facilitate the structural and functional interpretation of the reported complex compounds, the common name “phosphatidylperoxidiinylglycerol carboxymethylated carbomethylated” has been adopted for the compound identified in PubChem as 2-[2-[[(2R)-2,3-bis(docosa-9,11-diynylperoxy)propoxy]-hydroxyphosphoryl]oxyethyl-(carboxymethyl)amino]acetic acid (CID: 100946793). This designation highlights three key elements of its structure: (i) the phosphatidylglycerol skeleton, (ii) the presence of peroxidized acyl chains derived from docosa-9,11-diinoic acid, and (iii) an aminoacetate-like carboxymethylated polehead. The choice of the name is based on criteria of clarity, coherence with the functional nomenclature used in complex lipids, and biochemical relevance, avoiding ambiguities associated with extensive structural codes or technical acronyms that are difficult to interpret.

**Table 5:** Butanolic Fraction of *Piper marginatum*—Tentative Identification of Secondary Metabolites by LC-QTOF/MS.

t_R_ min	*m*/*z*			Adduct	Formula	Tentative Identification			Metabolite Class	References
	Exp	Theoric	Error ppm			Common Name	IUPAC Name	ID Level		
30.32	149.0226	166.0266	5	M+H−H_2_O	C_8_H_6_O_4_	Piperonylic acid	1,3-benzodioxole-5-carboxylic acid	3	Benzodioxol acid derivative	[132,133,134]
14.86	158.1529	157.1467	7	M+H	C_9_H_19_NO	Lastar A	2,2,6,6-tetramethyl-4-piperidinol	3	Alkaloid (piperidine derivative)	[135,136,137,138]
16.48	179.0699	178.0630	2	M+H	C_10_H_10_O_3_	Coniferyl aldehyde	(E)-3-(4-hydroxy-3-methoxyphenyl)prop-2-enal	3	Phenylpropanoid (cinnamaldehyde derivative)	[139,140,141]
18.38	179.1060	196.1099	4	M+H−H_2_O	C_11_H_16_O_3_	3-veratril-1-propanol	3-(3,4-dimethoxyphenyl)propan-1-ol	3	Phenylpropanoid (phenylpropane alcohol)	[142,143,144]
18.21	195.0660	194.0579	4	M+H	C_10_H_10_O_4_	Kakuol	1-(6-hydroxy-1,3-benzodioxol-5-yl)propan-1-one	3	Benzodioxol derivative	[145,146,147,148]
19.56	197.0802	196.0736	3	M+H	C_10_H_12_O_4_	Safrolglycol	3-(1,3-benzodioxol-5-yl)propane-1,2-diol	3	Benzodioxol derivative	[149,150,151,152]
18.38	197.1170	196.1099	1	M+H	C_11_H_16_O_3_	Loliolide	(6S,7aR)-6-hydroxy-4,4,7a-trimethyl-6,7-dihydro-5H-1-benzofuran-2-one	3	Benzofuranonic lactone	[153,154,155,156]
23.45	225.1115	224.1049	3	M+H	C_12_H_16_O_4_	Syringylacetone	4-(4-Hydroxy-3,5-dimethoxyphenyl)butan-2-one	3	Phenylpropanoids (cinnamic acid derivative)	[157,158,159,160]
24.18	225.1115	224.1049	3	M+H	C_12_H_16_O_4_	Pogostone/Dhelwangin	4-Hydroxy-6-methyl-3-(4-methylpentanoyl)-2H-pyran-2-one	3	Pyrone/polyketoid lactone	[161,162,163,164]
17.90	225.1116	224.1049	2	M+H	C_12_H_16_O_4_	Acoramone	1-(2,4,5-Trimethoxyphenyl)propan-2-one	3	Phenylpropanoid (keto-phenylpropane)	[165,166,167,168]
18.75	225.1117	224.1049	2	M+H	C_12_H_16_O_4_	Isoacoramone	1-(2,4,5-Trimethoxyphenyl)propan-1-one	3	Phenylpropanoid (keto-phenylpropane)	[165,168,169,170]
21.31	225.1117	224.1049	2	M+H	C_12_H_16_O_4_	Aspidinol	1-(2,6-dihydroxy-4-methoxy-3-methylphenyl)butan-1-one	3	Phenylpropanoid (keto-phenylpropane)	[171,172,173,174]
19.97	225.1118	224.1049	1	M+H	C_12_H_16_O_4_	Senkyunolide I	(3Z,6S,7S)-3-butylidene-6,7-dihydroxy-4,5,6,7-tetrahydro-2-benzofuran-1-one	3	Lactone	[175,176,177,178]
15.27	235.1810	234.1732	2	M+H	C_14_H_22_N_2_O	Lupanyl acid	3-(7,11-diazatricyclo[7.3.1.0~2,7~]tridecan-10-yl)propanoic acid	3	Triterpenoid derivative (lupanoid)	[72,73,74]
18.45	255.0679	272.0685	9	M+H−H_2_O	C_15_H_12_O_5_	Dihydrobaicalein	5,6,7-trihydroxy-2-phenyl-2,3-dihydrochromen-4-one	3	Flavonoid (flavanone)	[179,180,181,182]
20.51	274.2737	273.2668	1	M+H	C_16_H_35_NO_2_	C16 Sphinganine	(2S,3R)-2-aminohexadecane-1,3-diol	3	Sphingolipid (phytosphingolipid/ceramide base)	[183,184,185,186]
21.31	293.1357	292.1311	9	M+H	C_16_H_20_O_5_	Coriandrone B	3-hydroxy-5-methoxy-2,2,8-trimethyl-3,4,7,8-tetrahydropyrano[4,3-h]chromen-10-one	3	Benzopyran derivatives	[187,188,189,190]
24.59	304.2992	323.3188	4	M−H−H_2_O	C_21_H_41_NO	N-isopalmitoylpyrrolid *	14-methyl-1-pyrrolidin-1-ylhexadecan-1-one	3	Fatty acid-derived aliphatic amide	[191,192,193]
18.58	314.1384	313.1314	1	M+H	C_18_H_19_NO_4_	Feruloyl tyramine	(E)-3-(4-hydroxy-3-methoxyphenyl)-N-[2-(4-hydroxyphenyl)ethyl]prop-2-enamide	3	Phenylpropanoid amide (hydroxycinnamamide acid derivative)	[194,195,196,197]
20.65	318.2996	317.293	2	M+H	C_18_H_39_NO_3_	Phytosphingosine	(2S,3S,4R)-2-aminooctadecane-1,3,4-triol	3	Sphingolipid (phytosphingolipid/hydroxy-sphingosine)	[198,199,200,201]
18.46	326.1417	325.1373	9	M+H	C_12_H_23_NO_9_	Galactosyl-DNJ	(2S,3R,4S,5R,6R)-2-[(3S,4S,5R,6R)-4,5-dihydroxy-6-(hydroxymethyl)piperidin-3-yl]oxy-6-(hydroxymethyl)oxane-3,4,5-triol	3	Glucopyranosylated iminosaccharide	[202,203,204,205]
26.07	332.3305	349.3345	2	M+H−H_2_O	C_23_H_43_NO	Pipercitine	(E)-1-piperidin-1-yloctadec-2-en-1-one	3	Aliphatic azacycloalkane	[206,207,208,209]
27.93	593.2764	592.2686	1	M+H	C_35_H_36_N_4_O_5_	Pheophorbide A	3-[(3R,21S,22S)-16-ethenyl-11-ethyl-4-hydroxy-3-methoxycarbonyl-12,17,21,26-tetramethyl-7,23,24,25-tetrazahexacyclo[18.2.1.15,8.110,13.115,18.02,6]hexacosa-1,4,6,8(26),9,11,13(25),14,16,18(24),19-undecaen-22-yl]propanoic acid	3	Tetrapyrrole	[210,211,212,213]
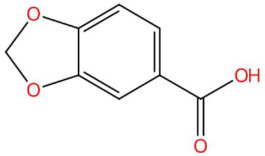						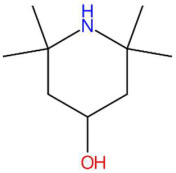		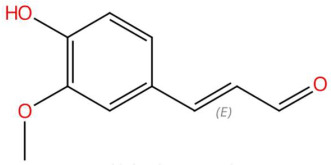		
Piperonylic acid (t_R_ 30.32 min; C_8_H_6_O_4_) 166.0266 Da						Lastar A (t_R_ 14.86 min; C_9_H_19_NO) 157.1467 Da		Coniferyl aldehyde (t_R_ 16.48 min; C_10_H_10_O_3_) 178.0630 Da		
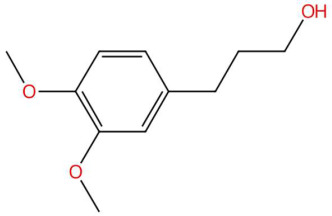						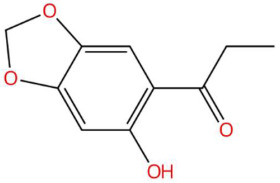		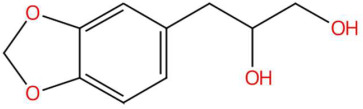		
3-veratril-1-propanol (t_R_ 18.38 min; C_11_H_16_O_3_) 196.1099 Da						Kakuol (t_R_ 18.21 min; C_10_H_10_O_4_) 194.0579 Da		Safrolglycol (t_R_ 19.56 min; C_10_H_12_O_4_) 196.0736 Da		
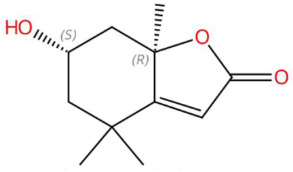						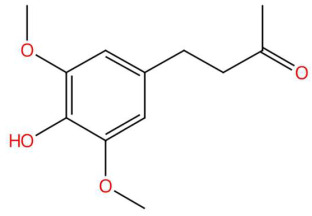		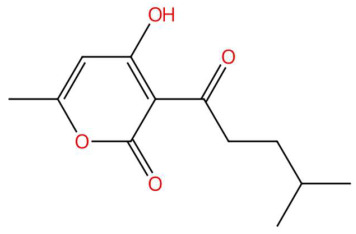		
Loliolide (t_R_ 18.38 min; C_11_H_16_O_3_) 196.1099 Da						Syringylacetone (t_R_ 23.45 min; C_12_H_16_O_4_) 224.1049 Da		Dhelwangin/Pogostone (t_R_ 24.18 min; C_12_H_16_O_4_) 224.1049 Da		
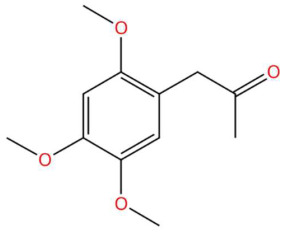						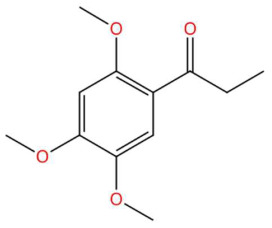		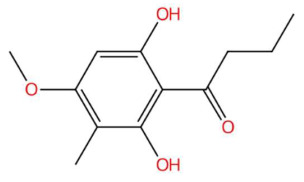		
Acoramone (t_R_ 17.90 min; C_12_H_16_O_4_) 224.1049 Da						Isoacoramone (t_R_ 18.75 min; C_12_H_16_O_4_) 224.1049		Aspidinol (t_R_ 21.31 min; C_12_H_16_O_4_) 224.1049 Da		
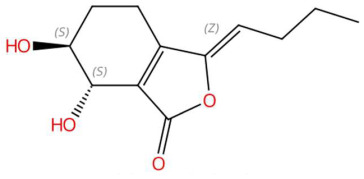						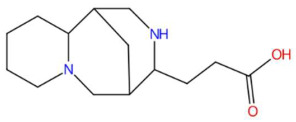		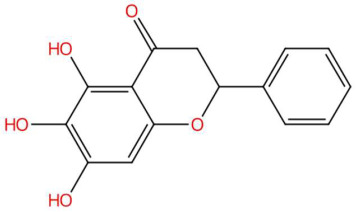		
Senkyunolide I (t_R_ 19.97 min; C_12_H_16_O_4_) 224.1049 Da						Lupanyl acid (t_R_ 15.27 min; C_14_H_22_N_2_O) 234.1732 Da		Dihydrobaicalein (t_R_ 18.45 min; C_15_H_12_O_5_) 272.0685 Da		
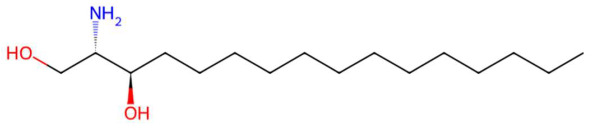						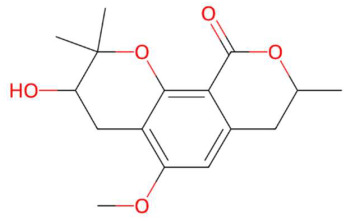		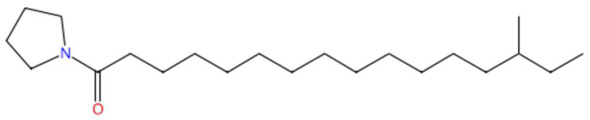		
C16 Sphinganine (t_R_ 20.51 min; C_16_H_35_NO_2_) 273.2668 Da						Coriandrone B (t_R_ 21.31 min; C_16_H_20_O_5_) 292.1311 Da		N-isopalmitoylpyrrolid (t_R_ 24.59 min; C_21_H_41_NO) 323.3188 Da		
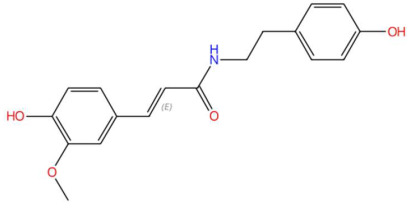						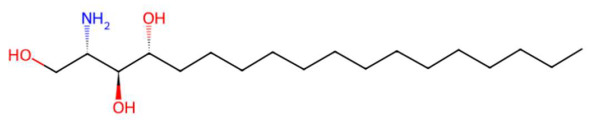		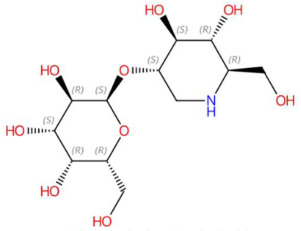		
Feruloyl tyramine (t_R_ 18.58 min; C_18_H_19_NO_4_) 313.1314 Da						Phytosphingosine (t_R_ 20.65 min; C_18_H_39_NO_3_) 317.293 Da		Galactosyl-DNJ (t_R_ 18.46 min; C_12_H_23_NO_9_) 325.1373 Da		
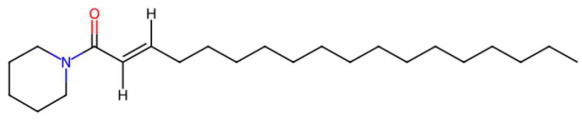						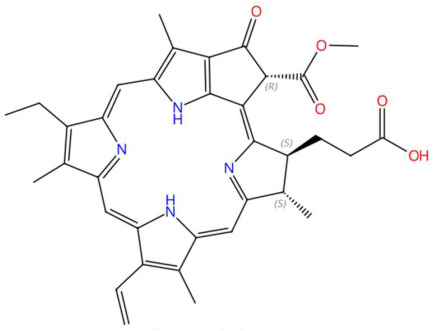				
Pipercitine (t_R_ 26.07 min; C_23_H_43_NO) 349.3345 Da						Pheophorbide A (t_R_ 27.93 min; C_35_H_36_N_4_O_5_) 592.2686 Da				

* To facilitate understanding and terminological consistency within the present manuscript, the common name N-isopalmitoylpyrrolidine has been adopted to refer to the compound systematically identified as 1-(14-methylhexadecanoyl)pyrrolidine (PubChem CID: 6430518). This designation was selected for three main reasons: (i) it maintains the widely accepted convention of designating amide derivatives of fatty acids by the prefix “N-acyl” followed by the name of the corresponding amine, (ii) it adequately recognizes the methyl branching at position 14 of the precursor fatty acid, which is consistent with the use of the term “isopalmitoyl” in the specialized literature, and (iii) it is more accessible and representative for readers working in the area of phytochemistry or metabolomics of natural products, where these types of compounds are usually described by trivial nomenclature based on their structural skeletons. This choice does not alter the structural precision of the compound and allows for a clearer communicative approach without sacrificing scientific rigor.

## Data Availability

The original contributions presented in the study are included in the article material.
